# Maternal and early‐life area‐level characteristics and childhood adiposity: A systematic review

**DOI:** 10.1111/obr.12861

**Published:** 2019-04-29

**Authors:** Sam Wilding, Nida Ziauddeen, Dianna Smith, Paul Roderick, Nisreen A. Alwan

**Affiliations:** ^1^ School of Primary Care and Population Sciences, Faculty of Medicine University of Southampton Southampton UK; ^2^ School of Geography and Environmental Science, Faculty of Environmental and Life Sciences University of Southampton Southampton UK; ^3^ NIHR Southampton Biomedical Research Centre University of Southampton and University Hospital Southampton NHS Foundation Trust Southampton UK

**Keywords:** children, DOHaD, environment, obesity

## Abstract

There is a cross‐sectional evidence that physical and social environments are linked to childhood adiposity. Evidence is scarce for the role of preconception, pregnancy, and early‐life area‐level characteristics in shaping childhood adiposity. We aimed to systematically review evidence for associations between physical and social environmental conditions experienced in these periods and childhood adiposity. Published literature was identified from the CINAHL, Embase, MEDLINE, and PsycINFO databases. Longitudinal studies linking an area‐level environmental exposure in the preconception, pregnancy, or early‐life (less than 1 year) periods and a measure of adiposity between the ages of 2 and 12 years were examined. Eight studies in the United States, Denmark, South Korea, United Kingdom, and Canada satisfied the inclusion criteria. Storm‐induced maternal stress, nitrogen oxides exposure, traffic noise, and proximity were associated with greater childhood adiposity. Frequent neighbourhood disturbances were associated with lower adiposity, while particulate matter exposure was associated with both higher and lower adiposity in childhood. Area‐level characteristics may play a role in the ongoing obesity epidemic. There is a limited evidence of longitudinal associations between preconception, pregnancy, and early‐life area‐level characteristics with childhood adiposity. Numerous factors that appear important in cross‐sectional research have yet to be assessed longitudinally, both individually and in combination.

## 1 INTRODUCTION

Overweight and obesity among children are growing global health concerns. In 2016, an estimated 50 million girls and 74 million boys aged 5 to 19 years were affected by obesity worldwide.^1^ Children affected by overweight and obesity are at risk of developing type 2 diabetes^2^ and are at higher risk of cardiovascular risk factors (high blood pressure and cholesterol) during adulthood.^3^ Early intervention is key, as childhood weight is strongly associated with adult weight.^4^ Inequalities are apparent in the prevalence of children with overweight and obesity between areas with differing socio‐economic and environmental characteristics.^5^, ^6^ In England, children living in the most socio‐economically deprived areas are more than twice as likely to be affected by obesity than children in the most affluent areas.^7^

Over the last 30 years or so, the Developmental Origins of Health and Disease (DOHaD) paradigm has identified the preconception, antenatal, and early‐life periods as key to shaping future susceptibility to non‐communicable diseases (NCDs).^8^ The circumstances experienced in these key phases of life enact epigenetic and behavioural adaptations among offspring, which have implications for their development and later health.^9^ The environment that mothers experience in the preconception period and those that their children are exposed to in‐utero and in their first year of life are likely to be important dimensions influencing later adiposity growth in childhood through numerous plausible mechanisms.

The characteristics of the physical environment influence dietary and physical activity habits that affect overall health and risk of mothers being affected by overweight or obesity at conception, and during pregnancy. Proximity to fast food outlets may encourage consumption of food that is of poor nutritional value.^10^ Mothers living within a half‐mile of a fast food restaurant are more likely to gain over 20 kg during pregnancy,^11^ and high gestational weight gain is a known risk factor for offspring being affected by obesity.^12^ Conversely, the lack of accessible healthy food (in so called “food deserts”) may also affect maternal diet in the preconception and pregnancy periods, with gestational undernutrition (as indicated by premature births and low birth weight) being linked with the risk for children to be affected by obesity through offspring compensatory growth post‐birth and increased leptin resistance.^13^, ^14^ Attractive open and green environments encourage women to walk in the prenatal and perinatal periods, enhancing physical activity and offering opportunities for social interaction that may alleviate stress.^15^ Stress during pregnancy has been linked with alterations in placental endocrine and immune processes, resulting in higher risk of infants being born premature and small for gestational age, which is associated with compensatory growth in early infancy and subsequent adiposity in childhood.^16^

Some environmental factors that affect childhood adiposity may be specific to the pregnancy period. Mothers exchange ingested and inhaled pollutants with offspring via placental transfer, which affects fetal and infant development.^17^ Gestational exposure to organic pollutants from indoor and outdoor sources has been shown to lead to elevated insulin and leptin levels, in addition to impaired glucose tolerance in rats, factors that affect the storage and expenditure of energy and therefore the risk of becoming affected by obesity.^18^ The diversity of maternal gut bacteria (the “microbiome”) affects nutrition exchange and the composition of the offspring microbiome at birth.^19^ The composition of the antenatal microbiome is influenced by maternal exposure to environmental pollutants, with particular combinations being associated with susceptibility to NCDs.^20^ For example, high counts of *Lactobacillius* bacteria in the antenatal microbiome are associated with high risk of offspring being affected by overweight and obesity during infancy and childhood.^21^

The environment and diet children are exposed to in their first year of life may also affect their weight during childhood. The diversity of gut microbiota is formed in the first few hours of human life as a response to the antenatal microbiome^22^ and rapidly evolves as a result of exposure to environmental pollutants,^20^ and the microbiome is associated with weight in childhood.^23^ The proximity of supermarkets and fast food outlets to the home and workplace is associated with dietary patterns among adults and hence families.^24^ Through breast milk, mothers exchange nutrients from food with infants^25^; this exchange develops infant familiarity and preference for the foods which mothers eat.^26^ In this way, the food environment at this stage can affect later childhood diet through post‐natal diet. Exposure to environmental allergens in the first year of life has been linked with lower risk of recurrent wheezing,^27^ which may make physical activity more feasible during childhood. Exposure to common air pollutants (sulphur dioxide, nitrogen dioxide, and particulate matter) during the first year of life hamper lung function, leading to respiratory problems in childhood,^28^ which may also affect physical activity patterns during childhood.

The creation of “health‐promoting environments” is one of the World Health Organization's objectives for preventing NCDs such as obesity.^29^ What exactly constitutes a health‐promoting environment is contested, and applications at the regional and national levels have been mixed. Preschools, schools, and deprived neighbourhoods are identified as environments that are conducive to obesity within the European Union 2014 to 2020 Action Plan, with no attention paid to the early‐life neighbourhood environment.^30^ The U.K. Government's action plan also identifies schools and early‐year settings as environments where children are exposed to obesity‐related risks, with no reference to the neighbourhood environment.^31^ In addition, neither framework acknowledges the role that the preconception environment plays in subsequent offspring health.

In previous systematic reviews, neighbourhood socio‐economic deprivation,^6^ parental perception of neighbourhood safety,^32^ fast food availability,^5^ access to open natural (green) spaces, and physical activity facilities^33^ were associated with childhood adiposity. The evidence base mostly consists of cross‐sectional studies; therefore, the extent to which the environment is causally associated with childhood adiposity is difficult to establish, as there is no information on the length of exposure to environmental influences.^34^ In the context of conflicting definitions of “health promoting environments,” and to inform policies that can target high‐risk neighbourhoods with preventive interventions, a comprehensive review is needed to collate the evidence on longitudinal associations between specific area‐level characteristics and childhood adiposity. Hence, the aim of this study is to systematically identify research which characterizes area‐level environmental exposures experienced in the preconception and antenatal periods as well as the first year of life and test their association with later childhood adiposity.

## 2 METHODS

### 2.1 Search strategy

A systematic search of published literature was conducted through searching the CINAHL, Embase, MEDLINE, and PsycINFO databases. The search strategy is detailed in Table S1. The final search was conducted on the 28th of August 2018, after consulting with a specialist librarian. Studies were limited to those published in English, and from January 1, 1990, to ensure that up‐to‐date literature was assessed. The reference list of all full‐texts that were included was searched. The protocol for this review was published on the PROSPERO international prospective register of systematic reviews (CRD42017082020), and this review is reported in line with the PRISMA guidelines.^35^

### 2.2 Inclusion and exclusion criteria

There were five main inclusion criteria in this review. Studies must be longitudinal, as we are interested in environmental exposure in the preconception, pregnancy and early‐life periods, and their associations with adiposity in childhood. Studies must have a measure of adiposity as the outcome. Measures of adiposity can include body mass index (BMI; kg/m^2^), weight‐for‐gestational‐age, bioelectrical impedance analysis, skinfold measurements, waist circumference, and body adiposity, where the methodology is justified. Cut‐offs for classifying children as being affected by overweight or obesity are also eligible, where the cut‐off is clearly defined and justified. The outcome must be measured in childhood (between 2 and 12 years old). Characteristics of the residential or workplace environment must be assessed through geo‐referencing, or be self‐reported. Environmental characteristics must be measured during the preconception, pregnancy, or early‐life (younger than 1 year old) periods. Studies where the sole outcome was change in adiposity were excluded, as a change in growth velocity may not result in a difference in adiposity when there are differences in birth and early‐life weight. Studies that used personal devices to monitor environmental features were also excluded, as measurements would have been affected by in‐home, neighbourhood, and out‐of‐neighbourhood features. Self‐reported measures were only eligible if they explicitly mention the residential or workplace neighbourhood, the surrounding area or the “local area.” Research published in non–peer‐reviewed or “grey” literature (including books, book chapters, conference proceedings, working papers, and theses) were also excluded due to the scale of peer‐reviewed papers retrieved in preliminary searches and the lack of quality control afforded by the exclusion of peer‐review in these outputs.^36^

### 2.3 Screening process

A 10% randomly selected sample of titles was screened for eligibility independently by two reviewers (S.W. and N.Z.) using Rayyan, a screening management software.^37^ The 10% threshold was used, as a simulation study has shown that there is no decrease in study selection bias if the sampling fraction is increased^38^ past 10%. The percentage agreement between the two reviewers was 94% at the title stage. Discrepant decisions for inclusion/exclusion were arbitrated by a third reviewer (N.A.A.), and then one author (S.W.) screened the remaining titles for inclusion. The titles screened for inclusion followed the same process for abstracts, with the agreement between reviewers standing at 100%. All full‐texts were screened independently by S.W. and N.Z., with disagreements resolved in a meeting with the two reviewers and N.A.A. At this stage, study authors were contacted for details of subgroup analyses if their age intervals for the exposure or outcome included ineligible ages, or for further clarity on exposure assessment. Two authors replied with no further data gained, and one author did not reply.

### 2.4 Data extraction

Data extraction was conducted for all final included articles by S.W. using a modified version of the Cochrane Collaboration's data extraction form.^39^ The fully adjusted association estimates between each eligible environmental indicator and outcome were extracted, including for all subgroup analyses. In cases where there were multiple time points, all age‐eligible associations were extracted. Significant associations were identified through confidence intervals that did not overlap the null, or *P* values < .05 if confidence intervals were not presented.

### 2.5 Quality assessment

Quality assessment was conducted by two reviewers (S.W. and N.Z.). All eligible articles were prospective cohort studies, and there is no agreed scoring criteria for such studies. As a result, we elucidated key strengths and weaknesses of each study using the National Institute of Health (NIH) Assessment Tool for Observational Cohort and Cross‐Sectional Studies and the STROBE checklist.^40^, ^41^ The exclusion of sample members born preterm or low birth weight was considered a key weakness in studies which looked at in‐utero exposure, because these outcomes may be on the causal pathway between the pregnancy environment and later childhood adiposity. This stance is informed by evidence that 13% to 24% of preterm births globally are attributable to PM_2.5_ exposure in a logistic regression model^42^ and that PM_2.5_ exposure increases the risk of being born low birth weight.^43^ Being born preterm or low birth weight subsequently affects childhood adiposity in turn through early‐life compensatory growth.^44^

### 2.6 Analysis and synthesis

As we expected significant variation in study design and environmental measures, a narrative synthesis was planned a priori, rather than a meta‐analysis approach. Environmental measures were grouped based on their similarity, and a summary of the effect sizes and precision is presented across each included study.

## 3 RESULTS

**Figure 1:**
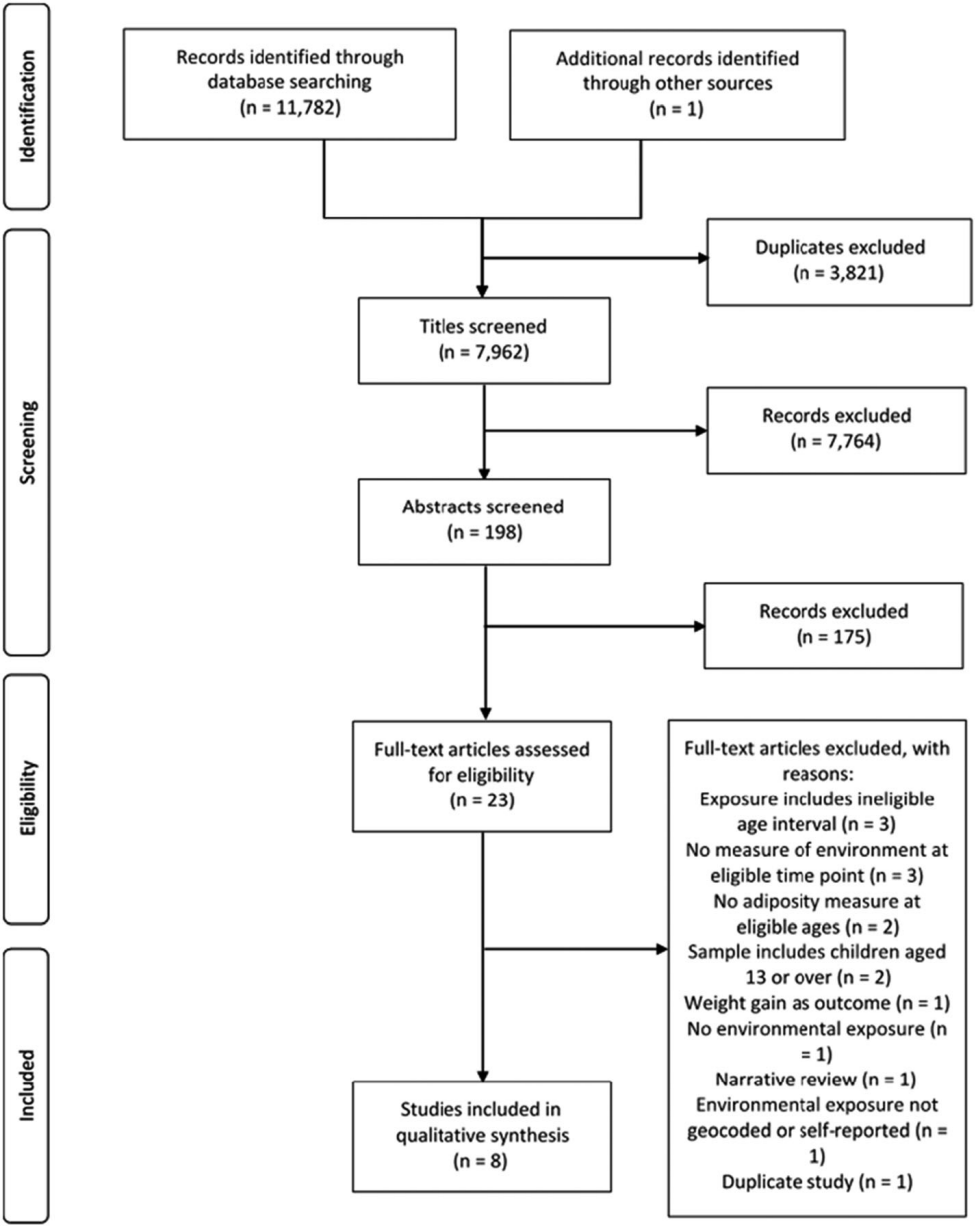
PRISMA flow diagram

A total of 11 783 records were identified in the search (Figure 1), of which 3821 were duplicates; 7962 titles were screened, of which 198 abstracts were further screened. A total of 23 full‐texts were assessed for inclusion independently. Two duplicate studies conducted by the same lead authors using the same dataset were identified,^45^, ^46^ one study was retained^45^ as environmental measures were included in the fully adjusted model, whereas they were not in the other study. In total, eight studies were included in the narrative synthesis.^45^, ^47^, ^48^, ^49^, ^50^, ^51^, ^52^, ^53^ Four studies were based in the United States, one each in Canada, Denmark, England, and South Korea. Seven of these studies were reports of a prospective cohort, and one was a secondary analysis of prospective cohort data.^45^ Generally, all studies were well reported and designed but had poor recruitment rates (less than 50%) or poor follow‐up rates (less than 80%). Further study characteristics are collated in Table 1.

**Table 1:** Study characteristics and quality assessment

Study	Design, Recruitment Rate, Follow‐Up Rate at First Outcome	Population, Setting	Exposure(s)	Age(s) at Exposure Measurement	Age(s) at Outcome Measurement	Outcome(s)	Quality Assessment^a^
Chiu et al^47^	Prospective cohort, 78% recruited, 77% followed up	247 children born ≥37 weeks gestation to English/Spanish speaking mothers, Boston (MA), US	PM_2.5_	Entire pregnancy	3‐5 years old	Age and sex‐ adjusted BMI z‐score, fat mass, waist‐to‐hip ratio	+ modelling technique estimates week‐specific effects + high recruitment rate − small sample size − exclusion of preterm births
Christensen et al^48^	Prospective cohort, 30% recruited, 55% followed up	54 968 children born ≥37 and <43 weeks gestation in Denmark	Road traffic noise, railway traffic noise	Entire pregnancy	7 years old	Age and sex‐ adjusted BMI z‐score, binary definition of overweight/obese or not based on BMI z‐score	+ repeated measures of exposure + large sample size − poor recruitment and follow‐up rate − exclusion of preterm births (although very rare)
Dancause et al^49^	Prospective cohort, 12% recruited, 83% followed up	116 children born to mothers experiencing a storm whilst pregnant, or 3 months before conception, Montérégie region, Canada	Timing of exposure to the storm, subjective stress related to storm (SR), objective stress related to storm (SR)	3 months preconception, first trimester, second trimester, third trimester	5 years old	Age and sex‐ adjusted BMI z‐score, binary definition of obese or not based on BMI z‐score	+ randomly‐distributed exposure + distinction between objective and subjective stress − small sample size, recruitment rate and follow‐up − over‐parameterised modelling
Fleisch et al^50^	Prospective cohort, 64% recruited, 68% followed up	1418 children, Boston‐area (MA), US	PM_2.5_, black carbon, traffic density, proximity to highway	Third trimester (PM_2.5_, black carbon), birth address (traffic density, proximity to highway)	2‐6 years old, 6‐10 years old	Age and sex‐ adjusted BMI z‐score, skinfold thickness, waist circumference, fat mass	+ subgroup analysis by age + distinction between sources of exposure (freeway and non‐freeway) − multiple outcome testing without *P* value adjustment − poor follow‐up rates
Hawkins et al^45^	Secondary analysis of prospective cohort, 72% recruited, 80% followed up	9184 children born between September 2000 and August 2001 in England	Access to food shops [SR], neighbourhood conditions [SR], satisfaction with area [SR], presence of safe play areas [SR], access to a garden [SR], indices of multiple deprivation	9 months old	3 years old	Binary definition of overweight/obese or not based on BMI z‐score	+ adjusted for early‐life (breast and solid) feeding + controls for migration between exposure and outcome − uses non‐validated self‐reported exposure measures − one exposure and outcome time‐point
Kim et al^51^	Prospective cohort, no information on recruitment rate, 37% followed up	1751 children born 2500 g + and at 37 weeks or more in Seoul, Cheonan or Ulsan, South Korea	PM_10_	Entire pregnancy, 0‐6 months, 7‐12 months	2 years old, 3 years old, 5 years old	Weight‐for‐age z‐score	+ repeated measures of exposure and outcome + adjusted for early‐life feeding method − poor follow‐up rate 1‐year after birth − exclusion of preterm births
Kim et al^52^	Prospective cohort, 65% recruited, 82% followed up	3424 public school‐children in Southern California, US	Nitrogen oxides (NO_x_), PM_2.5_	Entire pregnancy, first year	10 years old	BMI (kg/m^2^)	+ high recruitment rate and sample size + repeated exposure − extreme exposure measure (2 standard deviation difference) in models − unclear how missing data were handled
Mao et al^53^	Prospective cohort, no information on recruitment rate, 78% followed up	1446 singleton children born to mothers with a pre‐pregnancy BMI ≥18.5 and without a major birth defect, Boston (MA), US	PM_2.5_	Preconception (90 days before conception), first trimester, second trimester, third trimester, entire pregnancy	2‐5 years old, 6‐9 years old, 2‐9 years old	Binary definition of overweight/obese or not based on BMI z‐score	+ repeated exposure and outcome measures + assessed moderation by maternal pre‐pregnancy BMI − expected direction of association not clearly stated − no mention of addressing potential bias from over‐representation of urban, low‐income and minority ethnic mothers in sample

Abbreviations: BMI, body mass index (km/m^2^); SR, self‐reported.

a Quality assessed through an adapted version of the National Heart, Lung, and Blood Institute Quality Assessment Tool for Observational Cohort and Cross‐Sectional Studies and the STROBE checklist.^39^, ^40^

### 3.1 Characteristics of included studies

All eight included studies used data from prospective cohorts. Four studies recruited women during pregnancy,^47^, ^48^, ^50^, ^51^ one study recruited shortly after birth,^53^ one study used a combination of the two,^49^ one study recruited 9 months after birth,^45^ and one study recruited children through schools.^52^ The recruitment rate varied between 12%^49^ and 78%^47^ (mean 51%) and was not presented in two studies.^51^, ^53^ The percentage of the recruited sample who participated at each outcome time‐point varied between 22%^51^ and 83%^49^ (mean 60%).

The eight studies varied in terms of the timings of exposure and outcome measurement. Two studies had only one time‐point for exposure,^45^, ^48^ and four had only one time‐point for the outcome.^45^, ^47^, ^48^, ^49^ Five studies assessed the average exposure over the entire pregnancy,^47^, ^48^, ^51^, ^52^, ^53^ four across the first year of life,^45^, ^50^, ^51^, ^52^ and two in the preconception period, defined as 3 months prior to conception.^49^, ^53^ Three studies investigated trimester‐specific measures, including two studies that assessed the exposure in each trimester,^49^, ^53^ and one study that assessed exposure in the third trimester only.^50^

There were a total of eight anthropometric outcomes examined across the eight studies (BMI z‐score, weight‐for‐age z‐score, overweight or obesity based on BMI cut‐off, obesity based on BMI cut‐off, fat mass, waist‐to‐hip‐ratio, skinfold thickness, and waist circumference), with four studies examining more than one outcome.^47^, ^48^, ^49^, ^50^ All outcomes were measured by research assistants, school nurses, or GPs, with the exception of one study, where the measurer of height and weight was “undefined” for 35% of the sample.^48^ BMI was the most common outcome and was present in five studies (four used age‐ and sex‐adjusted z‐scores,^47^, ^48^, ^49^, ^50^ one did not^52^). An age‐ and sex‐ adjusted cut‐off for overweight/obesity was used in four studies,^45^, ^48^, ^49^, ^53^ and fat mass was assessed in two studies.^47^, ^50^ The following outcomes were assessed in only one study: waist‐to‐hip ratio^47^; waist circumference and skinfold thickness^50^; weight‐for‐age (ie, no height adjustment).^51^

A total of 16 environmental measures were tested across the eight included studies (10 measured through geo‐referencing and six self‐reported), of which seven were significantly associated with childhood adiposity in one or more studies (Table 2). Five groups of environmental indicators emerged from the review, which will be discussed in turn.

**Table 2:** Association between environmental indicators and childhood adiposity across eight studies

Measurement of Exposure	Environmental Measure	Chiu et al^47^	Christensen et al^48^	Dancause et al^49^	Fleisch et al^50^	Hawkins et al^45^	Kim et al^51^	Kim et al^52^	Mao et al^53^
Measured through geo‐referencing	Black carbon				×				
	Deprivation					×			
	Nitrogen oxides (NO_x_)							+	
	Noise from road traffic		+						
	Noise from railway traffic	+	×						
	Particulate matter (<2.5 μg/m^3^)				×			−	+
	Particulate matter (<10 μg/m^3^)						−		
	Traffic density				×				
	Traffic proximity				+				
	Trimester of exposure to a storm			×					
Self‐reported	Access to food					×			
	Frequency of neighbourhood disturbances					−			
	Garden access					×			
	Maternal stress related to a storm			+					
	Neighbourhood satisfaction					×			
	Safe play areas					×			

*Note*. (+) factor associated with greater childhood adiposity, (−) factor associated with reduced childhood adiposity, (×) confidence interval for association includes null (1.0 for odds ratios and relative risk ratios, 0.0 for linear coefficients), or if unavailable, *P* values > .05.

### 3.2 Air quality

Four air quality measures (black carbon, nitrogen oxides [NO_x_], and particulate matter [diameter < 2.5 μg/m^3^ and < 10 μg/m^3^]) were assessed across five studies at a variety of time points. Adjusting for maternal education, black carbon exposure during the third trimester was not associated with BMI z‐scores, skinfold fat, waist circumference, or fat mass in either early‐ (median age 3.3) or mid‐ (median age 7.7) childhood.^50^ Kim et al^52^ examined sources of NO_x_ exposure, and the association with adiposity, adjusting for maternal education and income. NO_x_ exposure from freeway sources averaged across the entire pregnancy was not associated with BMI at age 10, but there was an association for exposure in pregnancy among a subgroup who did not move home by 6 years of age (increase in BMI per 40.3 parts per billion NO_x_ 0.70 kg/m^2^, 95% CI, 0.07‐1.30). NO_x_ exposure from freeway sources averaged across the first year of life was associated with BMI at age 10 (increase in BMI per 39.1 parts per billion NO_x_ 0.50 kg/m^2^, 95% CI, 0.02‐0.90). There was no association for NO_x_ exposure from nonfreeway sources, but there was for total NO_x_ exposure in the first year of life (increase in BMI per 44.9 parts per billion NO_x_ 0.50 kg/m^2^, 95% CI, 0.02‐0.90).

Particulate matter less than 2.5 μg/m^3^ (PM_2.5_) was investigated in four studies and is the only environmental indicator explored in more than one study. Chiu et al examined the association between cumulative PM_2.5_ during pregnancy and adiposity at ages 3 to 5 separately for girls and boys, adjusting for maternal education. Among girls, there was no association with BMI z‐score or fat mass, but there was an association for waist‐to‐hip‐ratio (a 1 unit increase in cumulative PM_2.5_ [μg/m^3^] exposure during pregnancy was associated with an increase in waist‐to‐hip ratio of 0.02, 95% CI, 0.01‐0.03). Among boys, there was no association with waist‐to‐hip‐ratio, but there was an association for BMI z‐score (per μg/m^3^ cumulative PM_2.5_ exposure during pregnancy change in BMI z‐score 0.21, 95% CI, 0.00‐0.37) and fat mass (0.36 increase in BMI z‐score, 95% CI, 0.12‐0.68). Fleisch et al^50^ found no association between PM_2.5_ exposure in the third trimester and BMI z‐scores, skinfold fat, waist circumference, or fat mass in either early (median age 3.3) or mid‐ (median age 7.7) childhood, after adjusting for maternal education. Kim et al^52^ found that PM_2.5_ exposure was negatively associated with BMI at age 10, for exposure across pregnancy (per 17 parts per billion PM_2.5_ change in BMI −0.60 kg/m^2^, 95% CI, −1.10‐0.10) and across the first year of life (per 14.8 parts per billion PM_2.5_ change in BMI −0.50 kg/m^2^, 95% CI, −0.90 to −0.02), adjusting for maternal education. Mao et al found positive associations between greater quartiles of exposure to PM_2.5_ at preconception (90 days before pregnancy), all three trimesters and an average across trimesters, in regard to the risk of children being affected by overweight or obesity between the ages of 2 and 9 years, adjusting for maternal education and household income.

Kim et al^51^ found no association between PM_10_ exposure during pregnancy or from birth until 6 months with BMI z‐scores at ages 2, 3, and 5 years, adjusting for maternal education and income. There was an association between PM_10_ exposure during 7 to 12 months and BMI z‐score at ages 3 years (per 10 μg/m^3^ average exposure change in BMI z‐score −0.16, 95% CI, −0.37 to −0.05) and 5 years (−0.19, 95% CI, −0.34 to −0.06).

### 3.3 Traffic

Five traffic‐related measures were assessed in three studies. As discussed previously, Kim et al^52^ found that freeway‐NO_x_ exposure in‐utero was not associated with BMI at age 10, whereas greater exposure in the first year of life was positively associated with BMI. Christensen et al^48^ found that the average road traffic noise in‐utero was associated with the risk of these children being affected by overweight or obesity at age 7 (OR per 10 dB average 1.06, 95% CI, 1.00‐1.12), adjusting for maternal education and income. No association was present for rail traffic noise in the same study, however, and neither exposure was associated with BMI z‐scores.

Fleisch et al^50^ looked at traffic density and proximity at birth address for a range of adiposity outcomes (BMI z‐score, waist circumference, skinfold thickness, and fat mass) at ages 2 to 6 years and 6 to 10 years. There was no association between traffic density and any of the outcomes, adjusting for maternal education. For traffic proximity, there was a nonmonotonous association with adiposity, where the closest proximity (less than 50 m) and a further proximity (100‐200 m) were positively associated with BMI z‐scores, skinfold thickness and fat mass, compared with children furthest away (200 m+). The intermediate proximity category (50‐100 m) was not associated with any outcome, and there were no associations with waist circumference, skinfold thickness (at ages 6‐10 years) or fat mass (at ages 2‐6 years).

### 3.4 Social factors

Three area‐based social factors were assessed in one study based in England, after adjusting for maternal socio‐economic factors (social class, income, and education). Hawkins et al^45^ found that an area‐based measure of social and environmental deprivation (the 2015 Index of Multiple Deprivation^54^) at 9 months was not associated with the risk of children being affected by overweight or obesity at age 3 at two geographical levels with an average population size of 7000 and 1500, respectively (wards and Lower Super Output Areas [LSOAs]). The authors also utilized a question related to the frequency of neighbourhood disturbances (the examples given were noisy neighbours, rubbish/garbage, vandalism, and pollution) with responses coded in a 4‐point Likert scale from “not at all common” to “very common.” Very common poor neighbourhood conditions were associated with a lower risk of children being affected by overweight or obesity at age 3 (OR 0.73, 95% CI, 0.55‐0.98) relative to those responding “not at all common.” The neighbourhood satisfaction question used a similar Likert scale from “very dissatisfied” to “very satisfied” and was not associated with adiposity. Conversely, all of these measures were associated with the risk of mothers being affected by overweight or obesity in the same study.

### 3.5 Built environment

Hawkins et al^46^ also examined three subjective self‐reported measures of the local built environment (when the child was 9 months old), and their association with the risk of children being affected by overweight or obesity at age 3. Mothers were asked “how common are food shops and supermarkets that are easy to get to” [food access], with responses ranging between “not at all common” to “very common.” Mothers were also asked “are there any places where children can play safely” [safe play areas] and “do you have access to a garden” [garden access], with possible responses as yes or no to both questions. None of these measures of the built environment were associated with childhood adiposity.

### 3.6 Extreme weather conditions

Three measures of exposure to extreme weather events were examined by Dancause et al,^49^ adjusting for maternal socio‐economic status based on employment. In this study, women who were pregnant during or conceived in the 3 months following an ice storm in Canada were recruited, and their children were followed‐up at 5 ½ years old. The authors constructed three measures: (a) the trimester of exposure to the storm, (b) a scale of objective prenatal stress induced by the storm (eg, days without electricity, danger), and (c) a scale of subjective prenatal stress induced by the storm. A one‐point increase in the objective prenatal stress induced by the storm scale was associated with increased childhood BMI (β .22, *P* < .05) and risk of children being affected by overweight (OR 1.37, 95% CI, 1.06‐1.77, *P* .02), but there was no association for trimester of exposure nor subjective stress.

## 4 DISCUSSION

In this systematic review, eight studies were included that assessed longitudinal associations between certain preconception, pregnancy, or early‐life environmental factors and childhood adiposity. To our knowledge, this is the first review to systematically collate evidence on this subject.

In this review, five clusters of environmental measures emerged (in order of frequency): air quality, traffic, built environment, extreme weather conditions, and social factors. Associations with childhood adiposity were found within the first four, with the overall trend being in the expected direction in line with the Developmental Origins of Health and Disease (DOHaD) hypothesis.^55^ The exceptions to this trend were within the air quality cluster, with greater PM_2.5_ exposure in the first year of life being associated with lower BMI^52^ at age 10, and greater PM10 exposure in months 7 to 12 being associated with lower weight‐for‐age at 3 and 5 years.^51^ Also in the social factors cluster, common poor neighbourhood conditions at 9 months of age were associated with lower risk of children being affected by overweight or obesity^45^ at age 3. What these studies with surprising results have in common is timing of exposure assessment, as they assessed exposure in the first year of life. However, although Kim et al^52^ found that PM_2.5_ exposure in the first year of life was associated with lower BMI at age 10, the opposite was true for freeway NO_x_ in the same study.^52^ The two other factors (PM10 and neighbourhood conditions) were not evaluated in any other study, and therefore, further research is required to test the reproducibility of these findings. These three studies had limitations in their study design, with Hawkins et al utilizing only one exposure and outcome time point, Kim et al^51^ excluding preterm births and Kim et al^52^ using an extreme measure of exposure (per 2 standard deviation increase in exposure). On the other hand, all three studies had samples greater than 1500, increasing the power to detect such statistical associations.

The findings of this systematic review clearly display that there are important environmental exposures for which the longitudinal evidence on association with childhood adiposity is lacking, including socio‐economic deprivation,^6^ neighbourhood safety,^32^ and food access,^5^ all found to be linked to childhood adiposity in cross‐sectional research. Also, spaces for physical activity and green space were associated with childhood adiposity in a previous systematic review of cross‐sectional research^33^ but were not assessed in any studies in this review. The differences between the findings for longitudinal and cross‐sectional studies may be explained by residential sorting, where families with risk factors predisposing their children to being affected by obesity (eg, low‐income) may be more likely to move to disadvantaged neighbourhoods as their children grow,^56^ although adjustment for early‐life migration had no effect on estimates in Hawkins et al.^45^ Given that the above factors were not assessed across multiple studies in this review, further longitudinal research may shed greater light on these discrepancies.

Across the eight studies, geo‐referenced measures of the environment were more common than those which were self‐reported (Table 2). The results for self‐reported measures were in contrast to cross‐sectional research which has linked objective measures of the food environment^57^ and spaces for physical activity^58^ to childhood adiposity, although the evidence for these linkages is inconsistent.^59^, ^60^, ^61^, ^62^ Conversely, geo‐referenced area‐deprivation was not associated with childhood adiposity, despite cross‐sectional evidence.^6^ These findings suggest that there is some inconsistency in how these measures are measured between studies, or that these environmental characteristics bear different importance for childhood adiposity at various life‐course stages. Future work should compare geo‐referenced and self‐reported or subjective measures to better understand how these environmental characteristics may influence childhood adiposity.

All studies controlled for one or more individual measures of maternal socio‐economic status which is important to appropriately separate environmental and individual associations with health, because, for example, low‐income families, are more restricted in terms of dietary choices^63^ and are more likely to live in disadvantageous environments.^64^ All studies adjusted for either maternal smoking during pregnancy or maternal weight at various time points (with the exception of Kim et al^51^), factors that have been associated with offspring adiposity in previous research,^65^ improving the robustness of the studies reviewed herein. Only one study included measures of paternal smoking or weight,^45^ which is a limitation because paternal genetics, attitudes, and behaviours likely also affect childhood adiposity, and their exclusion from models overemphasizes the effect of maternal factors.^66^ The exclusion of preterm and low birthweight babies in some studies^47^, ^48^, ^51^ may have affected the estimates as these outcomes may be on the causal pathway between the environment and childhood adiposity, given evidence of links between the environment, birth outcomes,^42^ and infancy catch‐up growth for preterm and small babies.^44^, ^67^ Residual confounding may also occur at the area‐level, where certain environments experience multiple forms of disadvantage in terms of suitability for healthy weight gain, for example, areas with limited park access tend to have fewer outlets selling healthy foods.^68^ The results of these studies may have been influenced by area‐level confounding (in that the environmental measures are also correlated with unmeasured area characteristics which are directly associated with childhood adiposity), as only Christensen et al and Fleisch et al control for additional factors (urbanicity and neighbourhood income, respectively) at the area‐level.^48^, ^50^

All of the studies in this review used data from recruited prospective cohorts. Reliance on recruitment as opposed to routinely collected data may have led to sample bias affecting study estimates. Differences between the target population and the sample were noted in several studies,^48^, ^49^, ^50^ although Christensen et al noted that the effect of this bias was found to be insignificant in a comparison of cohort and register data.^69^ The lack of studies drawing on routine or administrative data is likely related to difficulties in attaining datasets where parental residential information is linked to childhood data. As all of the included studies were observational in nature, it is unclear whether the associations were causal. Dancause et al^49^ note, however, that the exposure in their study (exposure to a storm) was theoretically randomly distributed (with respect to socio‐economic status), suggesting that there is potential for a causal mechanism between extreme weather conditions, maternal stress, and childhood adiposity.

In addition to the potential bias arising from low response rates from specific subgroups, five of the included studies^48^, ^49^, ^50^, ^51^, ^53^ had attrition rates above 20%. Little to no effort was made to address the potential bias related to attrition or missing data, with Hawkins et al using survey‐derived weights and Fleisch et al^50^ imputing missing values for child ethnicity based on maternal ethnicity. All studies used complete‐case analysis, where participants are removed from analyses if they have incomplete data on either the outcome or covariates. This may have biased the results towards the null if particularly high‐risk groups were more likely to drop‐out from the cohort studies or to not respond to particular questions.^70^

The studies included in this review are limited to high‐income countries (Canada, Denmark, England, South Korea, and the USA), and this is likely to have influenced the scope of environmental factors included. The environmental factors examined in the included studies may be specific to high‐income countries. Individual risk factors for children being affected by obesity such as maternal pre‐pregnancy underweight, inadequate antenatal care, and protein‐calorie intake imbalance in pregnancy and childhood are more prevalent in low‐ and middle‐income countries and may have stronger influence than environmental factors in these contexts.^71^

All of the objective measures used in studies within this review have been assigned through the mother or child's home address, which will have resulted in faulty assumptions regarding activity spaces and environmental exposures. In the preconception and early pregnancy timeframes, mothers may commute to work, and through these journeys, they may have been exposed to differing environments than those experienced in the areas surrounding their home. For working mothers, this may lead to an underestimation of exposure, as areas surrounding workplaces have been found to be less socially advantaged and have higher densities of food outlets than residential neighbourhoods, for pre‐retirement adults and women specifically.^72^, ^73^ Similar error may occur in the first year of life, if infants are taken to different environments. Further work using GPS devices to track the time spent in various places such as a “daily path area,” which are then linked to the environmental characteristics of these places, can be used to attain a more accurate picture of individual exposure or activity spaces in future work.^74^

The limitations of this review are balanced by its strengths. The search was designed in conjunction with a librarian, and the search itself was conducted through several databases. A significant number of studies were retrieved from the search that limits the possibility that the search was too narrow. Although most studies were screened by one author (S.W.), agreement between two authors (S.W. and N.Z.) in a 10% random sample of results at the title and abstract screening stages was very high (94% and 100%, respectively). The majority of adiposity outcomes and environmental measures were objectively measured rather than self‐reported, eliminating the potential for reporting bias influencing the findings of the review. Finally, as the search was limited to studies that had at least a 1‐year time difference between measurement of exposure and outcome, temporal order is adhered to in each study.

Our search was limited to studies published in English, and there may be a wider literature published in other languages which would contribute to this review. The exclusion of grey literature may have biased the findings of this review towards significant or “non‐null” papers,^75^ especially given that all included papers presented at least one significant finding. This decision was made in the context of peer‐review acting as a quality control process for journal articles. There was no evidence that this exclusion led to a lack of papers with negative or “unexpected” findings, as there was disagreement between the findings in this review and the cross‐sectional literature for PM_2.5_ exposure, deprivation, and neighbourhood conditions. The lack of multiple studies for each environmental indicator limits the ability to summarize cumulative evidence. Few studies adjusted for area‐confounders, so we cannot isolate each specific environmental characteristic's influence on later childhood adiposity from the general milieu (which likely differs across the range of each indicator). The studies were located entirely in high‐income countries, so there is no evidence base to infer for middle‐ and low‐income contexts. There was no consensus on the best tool to score or grade observational cohort research, so in the interest of being objective, we were limited to listing the strengths and weaknesses that were elucidated using two commonly used checklists. This approach may be more transparent in understanding how studies were assessed and allow readers to self‐identify criteria which are important in their view.

Future research on the influence of preconception, pregnancy and early‐life area‐level factors on childhood adiposity is recommended, given the limitations of the included studies in this review. Objective measures of food access and neighbourhood conditions used in the cross‐sectional literature are missing from longitudinal studies of the environment and childhood adiposity. Also, there is a lack of research on how the combination of area‐level social and physical characteristics shape fetal and early‐life programming of later obesity risk, and how they interact with individual‐level factors. Longitudinal studies using representative population‐level data, in order to avoid bias in sample recruitment and attrition, are needed. Future analyses should test multiple area‐level indicators taking into account individual‐level confounders to elicit independent associations with childhood adiposity. Children who were born preterm or low birthweight should not be excluded, as these are potential mediators on the causal pathway between preconception and pregnancy environments and childhood adiposity.

## 5 CONCLUSION

In summary, six area‐level characteristics experienced during preconception, pregnancy, and early‐life showed associations with childhood adiposity in this review. Worse air quality and greater exposure to traffic in the preconception, in‐utero and early‐life periods, were associated with greater adiposity in childhood. Other factors such as area deprivation and garden access, significant in cross‐sectional research, were not associated with adiposity in longitudinal studies. This suggests that area factors may play a role in the ongoing obesity epidemic. However, numerous area‐factors which appear important in cross‐sectional research have yet to be assessed longitudinally. In addition, there is no evidence on the effects of multiple area‐disadvantage. Further research to ascertain the role of area‐level environment in the developmental origins of obesity is needed.

## CONFLICT OF INTEREST

No conflict of interest was declared.

## AUTHOR CONTRIBUTIONS

N.A.A. is the Principal Investigator of the project. All authors contributed to the conception and protocol of the review and have read and approved the final manuscript. S.W. was responsible for conducting the search, screening, quality assessment, and drafting the manuscript. N.Z. contributed to screening and quality assessment. N.A.A. contributed to screening.

## Supporting information

Table S1: Search strategies for the CINHAL, EMBASE, MEDLINE and PsycINFO databases
