# Supplementary material for: Maternal and early‐life area‐level characteristics and childhood adiposity: A systematic review
Source: Obes Rev. 2019 Apr 29;20(8):1093–105. doi: 10.1111/obr.12861 (PMC6612509; doi:10.1111/obr.12861)
Supplement: Supplementary file 1 — Table S1: Search strategies for the CINHAL, EMBASE, MEDLINE and PsycINFO databases [file OBR-20-1093-s001.docx]

Title: Maternal and early-life area-level characteristics and childhood adiposity: a systematic review

Authors: Sam Wilding, Nida Ziauddeen, Dianna Smith, Paul Roderick, Nisreen A Alwan

Corresponding author contact information: Dr Sam Wilding, [s.a.wilding@soton.ac.uk](mailto:s.a.wilding@soton.ac.uk). Room AC22, Public Health Sciences and Medical Statistics South Academic Block, Level C, Southampton General Hospital Tremona Road Southampton, UK SO16 6YD.

Supporting Information Table 1 (Table S1): Search strategies for the CINHAL, EMBASE, MEDLINE and PsycINFO databases

| **CINAHL, MEDLINE and PsycINFO via the EBSCO platform** | |
| --- | --- |
| Search number | Terms |
| **Concept 1** – *any measure of overweight/ obesity* | |
| 1 (free text in title or abstract) | Weight OR overweight OR "over weight" OR obes* OR "body mass index" OR BMI OR adipos* OR waist OR fat OR size OR overnutrition OR "over nutrition" OR "weight gain" OR "body composition" |
| 2 (MeSH) | "Weight gain" OR "Body weight" OR "Overweight+" OR "Obesity, abdominal" OR "Body mass index" OR "Body fat distribution+" OR "Waist-height ratio" OR "Overnutrition" OR "Body composition+" |
| 3 | (Search 1 OR search 2) AND DT >=1990/01/01 |
| **Concept 2** – *measurement during childhood* | |
| 4 (free text in title or abstract) | Child* OR youth# OR young OR youngster# OR kid# OR boys OR girls OR "school-age*" OR "school age*" OR "pre-school" OR "pre school" OR p#ediatric OR toddler |
| 5 (MeSH) | "Child+" |
| 6 | (Search 4 OR search 5) AND DT >=1990/01/01 |
| **Concept 3** – *residential or workplace environment* | |
| 7 (free text in title or abstract) | Environment* OR obesogenic* OR community OR neighbo#rhood OR poverty OR deprivation OR deprived OR "food outlet#" OR "food store#" OR grocer* OR supermarket# OR restaurant# OR "urban design" OR "urban planning" OR walkable OR walkability OR playground* OR "playing field" OR park# OR "land-mix" OR "land mix" OR "land use" OR greenspace# OR "green space#" OR bluespace# OR "blue space#" OR "natural space#" OR forest# OR noise OR "air quality" OR "air pollution" OR "air pollutant#" |
| 8 (MeSH) | "Poverty areas" OR "Social environment+" OR "Social conditions" OR "Cultural deprivation" OR "Small-area analysis" OR "Residence characteristics" OR "Environment and Public Health" OR "Noise+" OR "Air pollution+" OR "Environmental exposure" OR "Air pollutants" OR "Gardens" OR "Restaurants" OR "Sports and recreational facilities+" OR "Transportation facilities+" OR "particulate matter" OR "Vehicle emissions" OR "Food services" OR "Health services accessibility" OR "City planning" OR "Environment design" |
| 9 | (Search 7 OR search 8) AND DT >=1990/01/01 |
| **Concept 4** – *measurement (of the environment) before or during pregnancy, or the first year of the child’s life* | |
| 10 (free text in title or abstract) | Pregnan* OR prenatal OR "pre-natal" OR preconception OR “pre-conception” OR antenatal OR "ante-natal" OR perinatal OR "peri-natal" OR birth OR "first year" OR "first-year" OR "early life" OR "early-life" OR infan* |
| 11 (MeSH) | "Perinatology" OR "Pregnancy" OR "Prenatal Care" OR "Beginning of Human Life" OR "Infant+" OR "Prenatal exposure delayed effects" OR "Maternal exposure" |
| 12 | (Search 10 OR search 11) AND DT >=1990/01/01 |
| 13 | Search 3 AND search 6 AND search 9 AND search 12 |
| **EMBASE Week 35 via the OVID platform** | |
| **Concept 1** – *any measure of overweight/ obesity* | |
| 1 (free text in title or abstract) | Weight OR overweight OR "over weight" OR obes* OR "body mass index" OR BMI OR adipos* OR waist OR fat OR size OR overnutrition OR "over nutrition" OR "weight gain" OR "body composition" |
| 2 (MeSH) | Weight gain |
| 3 (MeSH) | Body weight |
| 4 (MeSH) | Exp obesity |
| 5 (MeSH) | Exp body mass |
| 6 (MeSH) | Exp body fat distribution |
| 7 (MeSH) | Exp waist-height ratio |
| 8 (MeSH) | Overnutrition |
| 9 (MeSH) | Exp body composition |
| 10 (MeSH) | Search 1 OR search 2 OR search 3 OR search 4 OR search 5 OR search 6 OR search 7 OR search 8 OR search 9 |
| 11 (MeSH) | Limit search 10 to “1990-Current” |
| **Concept 2** – *measurement during childhood* | |
| 12 (free text in title or abstract) | (Child* or youth$ or young or youngster$ or kid$ or boys or girls or "school-age*" or "school age*" or "pre-school" or "pre school" or p$ediatric or toddler).ab,ti. |
| 13 (MeSH) | Exp child |
| 14 (MeSH) | Search 12 OR search 13 |
| 15 (MeSH) | Limit search 14 to “1990-Current” |
| **Concept 3** – *residential or workplace environment* | |
| 16 (free text in title or abstract) | (Environment* or obesogenic* or community or neighbo$rhood or poverty or deprivation or deprived or "food outlet$" or "food store$" or grocer* or supermarket# or restaurant$ or "urban design" or "urban planning" or walkable or walkability or playground* or "playing field" or park$ or "land-mix" or "land mix" or "land use" or greenspace$ or "green space$" or bluespace$ or "blue space$" or "natural space$" or forest$ or noise or "air quality" or "air pollution" or "air pollutant$").ab,ti. |
| 17 (MeSH) | Exp social environment |
| 18 (MeSH) | Cultural deprivation |
| 19 (MeSH) | Small-area analysis |
| 20 (MeSH) | Exp social medicine |
| 21 (MeSH) | Exp noise |
| 22 (MeSH) | Exp air pollution |
| 23 (MeSH) | Environmental exposure |
| 24 (MeSH) | Air pollutant |
| 25 (MeSH) | Exp “land use” |
| 26 (MeSH) | Catering service |
| 27 (MeSH) | Exp “traffic and transport” |
| 28 (MeSH) | Particulate matter |
| 29 (MeSH) | Exhaust gas |
| 30 (MeSH) | City planning |
| 31 (MeSH) | Environmental planning |
| 32 | Search 16 OR search 17 OR search 18 OR search 19 OR search 20 OR search 21 OR search 22 OR search 23 OR search 24 OR search 25 OR search 26 OR search 27 OR search 28 OR search 29 OR search 30 OR search 31 |
| 33 | Limit search 32 to “1990-Current” |
| **Concept 4** – *measurement (of the environment) before or during pregnancy, or the first year of the child’s life* | |
| 34 (free text in title or abstract) | (Pregnan* or prenatal or "pre-natal" or preconception or "pre-conception" or antenatal or "ante-natal" or perinatal or "peri-natal" or birth or "first year" or "first-year" or "early life" or "early-life" or infan*).ab,ti. |
| 35 (MeSH) | Perinatology |
| 36 (MeSH) | Pregnancy |
| 37 (MeSH) | Prenatal care |
| 38 (MeSH) | “Beginning of human life” |
| 39 (MeSH) | Exp infant |
| 40 (MeSH) | Prenatal exposure |
| 41 (MeSH) | Maternal exposure |
| 42 | Search 34 OR search 35 OR search 36 OR search 37 OR search 38 OR search 39 OR search 40 OR search 41 OR search 42 |
| 43 | Limit search 43 to “1990-Current” |
| 44 | Search 11 AND search 15 AND search 33 AND search 44 |
